# The cannabinoid Δ^9^-tetrahydrocannabivarin (THCV) ameliorates insulin sensitivity in two mouse models of obesity

**DOI:** 10.1038/nutd.2013.9

**Published:** 2013-05-27

**Authors:** E T Wargent, M S Zaibi, C Silvestri, D C Hislop, C J Stocker, C G Stott, G W Guy, M Duncan, V Di Marzo, M A Cawthorne

**Affiliations:** 1 Clore Laboratory, University of Buckingham, Buckingham, UK; 2 Endocannabinoid Research Group (ERG), Institute of Biomolecular Chemistry, Consiglio Nazionale delle Ricerche, Pozzuoli, Italy; 3 GW Pharmaceuticals, Porton Down Science Park, Salisbury, UK

**Keywords:** cannabinoid, DIO mice, *ob/ob* mice, insulin sensitivity, energy balance

## Abstract

**Background:**

Cannabinoid type-1 (CB1) receptor inverse agonists improve type 2 diabetes and dyslipidaemia but were discontinued due to adverse psychiatric effects. Δ^9^-Tetrahydrocannabivarin (THCV) is a neutral CB1 antagonist producing hypophagia and body weight reduction in lean mice. We investigated its effects in dietary-induced (DIO) and genetically (*ob/ob*) obese mice.

**Methods:**

We performed two dose-ranging studies in DIO mice; study 1: 0.3, 1, 2.5, 5 and 12.5 mg kg^−1^, oral twice daily for 30 days and study 2: 0.1, 0.5, 2.5 and 12.5 mg kg^−1^, oral, once daily for 45 days. One pilot (study 3: 0.3 and 3 mg kg^−1^, oral, once daily) and one full dose-ranging (study 4: 0.1, 0.5, 2.5 and 12.5 mg kg^−1^, oral, once daily) studies in *ob/ob* mice for 30 days. The CB1 inverse agonist, AM251, oral, 10 mg kg^−1^ once daily or 5 mg kg^−1^ twice daily was used as the positive control. Cumulative food and water intake, body weight gain, energy expenditure, glucose and insulin levels (fasting or during oral glucose tolerance tests), plasma high-density lipoprotein and total cholesterol, and liver triglycerides were measured. HL-5 hepatocytes or C_2_C_12_ myotubes made insulin-resistant with chronic insulin or palmitic acid were treated with 0, 1, 3 and 10 μℳ THCV or AM251.

**Results:**

THCV did not significantly affect food intake or body weight gain in any of the studies, but produced an early and transient increase in energy expenditure. It dose-dependently reduced glucose intolerance in *ob/ob* mice and improved glucose tolerance and increased insulin sensitivity in DIO mice, without consistently affecting plasma lipids. THCV also restored insulin signalling in insulin-resistant hepatocytes and myotubes.

**Conclusions:**

THCV is a new potential treatment against obesity-associated glucose intolerance with pharmacology different from that of CB1 inverse agonists/antagonists.

## Introduction

The clinical evidence for the efficacy of inverse agonists of the cannabinoid type-1 (CB1) receptor for the improvement of the metabolic status of animals with metabolic syndrome, type 2 diabetes and dyslipidaemia has grown over the past decade and is now widely recognized. However, the withdrawal of one such compound, Rimonabant, from the market in Europe in 2008 due to adverse psychiatric effects,^1^ led to a rapid interruption of pharmaceutical research in this field, with most of the large pharmaceutical companies abandoning the development of CB1 inverse agonists.

However, there remains much debate as to whether the safety issues observed with Rimonabant are related to its inverse agonism at the CB1 receptor or its penetration into the brain.^2^ In this sense, the development of compounds that are ‘neutral antagonists' of the CB1 receptor, that is, devoid of any action in tissues in which the receptor is constitutively coupled to G proteins, and in the absence of elevated levels of endogenous ligands, has shown promise. In fact, compounds that in functional assays *in vitro* exhibit neutral antagonism at CB1 receptors seem to possess different activity from inverse agonists also in *in vivo* assays.^3^ Whether such compounds show differential effects in humans also, while retaining clinical efficacy, remains to be demonstrated. Furthermore, it is still not well understood whether some of the beneficial metabolic effects (that is, reduction of glucose intolerance, dyslipoproteinaemia and hypertriglyceridaemia) of Rimonabant as well as of neutral CB1 antagonists^2, 4^ are merely because of the concomitant reduction of body weight or direct actions on peripheral tissues such as the adipose tissue, pancreas and skeletal muscle.^5^

It has been suggested that activation of cannabinoid type-2 (CB2) receptors, while not being overtly involved in those affective disorders that are often worsened by CB1 inverse agonism, improves glucose tolerance after a glucose load.^6^ On the other hand, more recent data with CB2 knockout mice^7, 8^ or mice overexpressing CB2 receptors in the brain^9^ have led to the opposite conclusion, although these results may have been confounded in part by concurrent changes in CB1 expression levels in metabolically active tissues. Thus, the role of CB2 receptors in the control of glucose metabolism is still under debate, and it is not clear yet whether agonists or antagonists at these receptors may produce beneficial metabolic effects.

Δ^9^-Tetra-hydrocannabivarin (THCV) is a naturally occurring analogue of the psychoactive principle of cannabis, Δ^9^-tetra-hydrocannabinol (THC). However, unlike THC, which is an agonist at cannabinoid CB1 and CB2 receptors, THCV, and its synthetic isomer Δ^8^-tetra-hydrocannabivarin, behaves as neutral CB1 antagonists and, depending on the *in vitro* and *in vivo* assays used, CB2 agonists or antagonists.^10, 11, 12, 13^ Importantly, THCV, like CB1 receptor antagonists/inverse agonists, and unlike THC, was found to produce hypophagic effects in both fasted and non-fasted mice.^14^ However, this compound has never been tested in obese rodents, and its potential beneficial effects on metabolic disturbances accompanying obesity, such as hyperglycaemia, dyslipidaemia and fatty liver, have never been evaluated. Here we present, for the first time, results from *in vivo* studies of the metabolic effects of THCV in dietary-induced obese (DIO) and genetically obese (*ob/ob*) mice.

## Materials and methods

### Animal studies

All mice were housed under controlled lighting conditions (12:12 h of light:dark, lights on at 0700 h) at a room temperature of 21±1 °C. All studies were conducted in accordance with UK Government Animals (Scientific Procedures) Act 1986 and approved by the University of Buckingham Ethical Committee.

For studies 1 and 2, female C57Bl/6 mice were obtained from Harlan Olac, Bicester, UK at age 5/6 weeks and placed on a high-fat diet containing 42% fat by kcal, 21.4% by weight (Western RD diet, Special Diet Services, Essex, UK). For study 1, mice received the diet for 30 weeks before the study, and for 19 weeks in study 2.

For studies 3 and 4, C57Bl/6 female *ob/ob* mice aged 5/6 weeks were obtained from Harlan Olac and placed on a chow diet (Bantin and Kingman no 1 rat and mouse diet, Hull, UK).

Before allocation to treatment, mice were weighed and a blood sample was taken to measure glucose. Mice were allocated to treatment groups (three cages of three mice per treatment in studies 1 and 2 for the DIO mice and two cages of four mice per treatment for the *ob/ob* mice) so that mean and s.d. of body weights and baseline glucose concentrations were similar across treatments.

For the twice daily dosing study (study 1), mice were dosed by gavage commencing at 1700 h on day 1 and thereafter at 0900 and 1700 h for 30 days. For the once daily dosing studies (studies 2–4), mice were dosed by gavage at 0900 h for 45 days in study 2, for 35 days in study 3 and for 30 days in study 4.

THCV (GW Pharmaceuticals, Salisbury, UK) was supplied as a stock solution in ethanol (472.6 mg ml^−1^). For studies 1–3, dose levels were appropriately diluted with sesame seed oil (S.I.O., Saint Laurent Blangy, France). The dose level of the formulated drug was 10 ml kg^−1^ with the ethanol concentration being 0.25 ml kg^−1^. Mice in the control group were given the sesame seed oil vehicle containing 2.5% ethanol. AM251 (Tocris, Bristol, UK) was dosed in the same vehicle. For study 4, the vehicle for dosing was 10% gelucrire 44/14 (Gattefosse, Lyon, France). For oral glucose tolerance tests (OGTTs), mice were fasted for 5 h before a 3-g kg^−1^ oral glucose load. Insulin was measured in plasma by enzyme-linked immunosorbent assay (Crystal Chem Inc, Downers Grove, IL, USA). Other analytes in blood or plasma were measured using 96-well assays (see Supplementary Methods). Energy expenditure was measured by open-circuit indirect calorimetry with mice in their home cages. Body composition was determined by DEXA scanning (Piximus, GE Medical Systems, Fitchburg, WI, USA).

### Biochemical analyses on blood

Blood samples were taken from the cut tip of the tail after the application of Lignocaine gel (Biorex Laboratories, Enfield, UK).

### Plasma preparation

Blood was collected in EDTA-coated microvettes (Sarstedt, Nümbrecht, Germany) for the measurement of plasma insulin, cholesterol or triglyceride concentration and stored on ice before centrifugation at 500 *g* for 5 min. The resulting plasma was stored at −80 °C until required.

### Blood glucose analysis

Samples of blood (10 μl) were taken into disposable micropipettes (Dade Diagnostics Inc., Aguada, Puerto Rico) and glucose concentrations were determined after mixing with 0.39 ml of haemolysis reagent. Duplicate 20 μl aliquots of this mixture was taken for each individual sample and placed in a 96-well assay plate. To each well was added 180 μl aliquots of glucose oxidase reagent (ThermoTrace, Noble Park, VIC, Australia), the samples were mixed and then left for ∼30 min. Samples were then analysed automatically using SpectraMax 250 and SoftMax Pro software (Molecular Devices Corporation, Sunnyvale, CA, USA).

### OGTT

Five hours before the start of the glucose tolerance test (0700 hours), food was removed and animals were given clean cages. Mice were treated with vehicle or THCV at 1130 hours and glucose at 1200 hours. Glucose was dissolved in water (3 g per10 ml) and orally given to the mice at a rate of 3 g kg^−1^. Blood samples (10 μl) were taken for the analysis of glucose concentration at −30, 0, 30, 60, 120 and 180 min following glucose administration. Blood samples were also taken at −30 and +30 min for insulin analysis. Food was returned at the end of the tolerance test.

### Plasma insulin analysis

Blood samples for the measurement of plasma insulin concentrations were taken from fed, 5-h fasted or overnight fasted mice. Plasma insulin was measured using 5 μl of plasma compared with a mouse insulin standard using a 96-well microassay plate (Crystal Chem Inc).

### Plasma triglyceride analysis

Blood samples were taken from fed mice for the analysis of plasma triglycerides. Samples of plasma (2 μl) were measured into a 96-well assay plate. To each well was added 200 μl aliquot of triglyceride reagent (ThermoTrace). The samples were mixed and then left for ∼45 min before measurement and analysed automatically using a SpectraMax 250 as above.

### Plasma cholesterol analysis

Blood samples were taken from fed mice for the analysis of plasma cholesterol. Plasma cholesterol was measured using 2 μl of plasma in a 96-well assay plate. To each sample was added 200 μl of infinity cholesterol liquid stable reagent (ThermoDMA, Louisville, CO, USA). The samples were mixed and incubated for 5 min before reading at 500 nℳ. The results were converted into cholesterol values using cholesterol standard (ThermoTrace) and SoftMax Pro software as above.

### Plasma high-density lipoprotein (HDL) cholesterol

Blood samples were taken from fed mice for analysis of HDL cholesterol. B-lipoprotein antibody binds to lipoproteins (low-density lipoprotein (LDL) cholesterol, very low-density lipoprotein cholesterol and chylomicrons) other than HDL. The antigen–antibody complexes formed block the action of cholesterol esterase so that only HDL cholesterol is available for assay by the standard cholesterol assay procedure (Trinity-EZ-HDL-Cholesterol, Trinity Biotech, Jamestown, NY, USA).

Plasma (1 μl) is added to 50 μl of reagent 1, which contains the β-lipoprotein antibody, and then, 150 μl of reagent 2 is added. This contains cholesterol esterase and cholesterol oxidase, which interact with HDL cholesterol to form hydrogen peroxide that in turn, in the presence of *N*-ethyl-*N*(2-hydroxy-3-sulphopropyl)-3,5-dimethoxy-4-fluoroalanine,4-aminoantipyrine and peroxidase, yields a blue colour complex that is measured at 600 nℳ in a Spectromax 250 plate reader.

The samples were incubated for 1 h at 37 °C.

### Liver triglycerides

The liver was removed from overnight fasted mice at the end of the study. Samples of liver (150–300 mg) were homogenized in 500 μl of methanol using a ribolyser cell disruptor at 4 °C. Chloroform (1 ml) was added, and tubes vortexed and left at 4 °C for 2 h with vortexing every 30 min. In all, 200 μl of 0.9% sodium chloride was added and after thorough vortexing the mixture is centrifuged at 300 *g* for 5 min. A 500-μl aliquot of the chloroform phase was taken and chloroform removed by evaporation. The residue was dissolved in 200 μl ethanol and triglyceride content measured.

### Energy expenditure measurements

Energy expenditure was measured by open-circuit indirect calorimetry^15^ with mice in their home cages, that is, energy expenditure was recorded on a group of mice. For studies of 24-h (days 9–10) energy expenditure, mice were dosed with their allocated treatment and then measurements commenced.

### *In vitro* experiments in hepatocytes and myocytes

HHL-5 cells were cultured in standard growth media, Dulbecco's modified Eagle's medium (DMEM; Lonza, Basel, Switzerland, supplemented with 10% fetal bovine serum (FBS), non-essential amino acids (NEAA) and penicillin streptomycin (Gibco, Paisley, Scotland). Two types of experiments were carried out with these human hepatocytes, which express CB1 receptor mRNA and nearly undetectable amounts of CB2 receptor mRNA, as determined by quantitative PCR (data not shown). (1) Experiments in insulin-sensitive cells were conducted as follows: cells were grown in six-well plates to 90% confluence and then cultured without (−) or with (+) THCV at the indicated concentrations for 24 h before being switched to serum-free media with or without THCV for 2 h, followed by stimulation with 100 nℳ insulin for 15 min. (2) Experiments in cells made insulin-resistant were carried out in two different ways: (i) cells in six-well plates were grown to 90% confluence and then treated with 250 μℳ palmitic acid or 100 nℳ insulin for 72 h in the absence (−) or presence (+) of THCV at the indicated concentrations for the final 24 h before being switched to serum-free media containing either 250 μℳ palmitic acid or vehicle with or without THCV for 2 h followed by stimulation with 100 nℳ insulin for 15 min (‘reversal' experiments); (ii) alternatively, cells in six-well plates were treated with 250 μℳ palmitic acid for only 24 h, which is still sufficient to induce insulin resistance, in the absence (−) or presence (+) of THCV or AM251 at the indicated concentration before being switched to serum-free media containing either 250 μℳ palmitic acid or vehicle with or without THCV or AM251 for 2 h followed by stimulation with 100 nℳ insulin for 15 min (‘prevention' experiment).

C_2_C_12_ cells were cultured in growth media DMEM supplemented with 10% FBS. To induce myotube formation, cells were grown to 90% confluence in six-well plates and then switched to differentiation media DMEM supplemented with 1% FBS, penicillin/streptomycin and amphotericin B (Sigma, St Louis, MO, USA) for 3 days before treatment with 250 μℳ palmitic acid for only 24 h, in the absence or presence of THCV or AM251 at the indicated concentrations before being switched to serum-free media containing 250 μℳ palmitic acid, with or without THCV or AM251 for 2 h followed by stimulation with 100 nℳ insulin for 15 min.

For analysis of protein levels, cells lysed in 1 × TNE buffer (50 mℳ Tris pH 7.4, 150 mℳ NaCl and 1 mℳ EDTA) with 1% Triton X-100, protease and phosphatase inhibitor cocktails (Sigma, Dorset, UK). Protein concentrations were analysed using Lowry protein assay (Bio-Rad, Hemel Hempstead, UK) to allow even protein loading for SDS–polyacrylamide gel electrophoresis using a continuous routine Tris-Glycine buffering system. Proteins transferred onto PVDF membranes were then blocked in 5% skim milk in TBST (20 mℳ Tris, 137 mℳ NaCl, 0.1% Tween-20) and probed with anti phospho-AKT (1:2000), AKT (1:1000, Cell Signaling Technology Inc, Danvers, MA, USA), overnight, followed by a HRP-conjugated secondary antibody (Bio-Rad) and then detected by ECL (Bio-Rad) and autoradiography. Band intensity quantification was performed with Image J (NIMH, Bethesda, MA, USA). Palmitic acid solutions for cell treatments were prepared from a stock solution (750 nℳ) in dimethyl sulphoxide (DMSO), which was then diluted to 5 mℳ in 10% bovine serum albumin in DMEM via sonication and incubation at 60 °C before being added to cell culture media to a final concentration of 250 μℳ. Bovine serum albumin solutions with DMSO alone were similarly prepared as for controls.

### Statistical methods

Results given in the text and data points in the figures are shown as the mean±s.e.m. The statistical significance of any differences between control animals and treated animals was determined using analysis of variance (ANOVA) tests followed by Dunnett's test. Statistical significance compared with data from the group given vehicle alone is shown as: *P<0.05, **P<0.01 or ***P<0.001. The order of the symbols on each graph is identical with the order on the key.

## Results

### Studies in DIO mice

#### Study 1 (twice daily dosing) and study 2 (once daily dosing)

**Figure 1:**
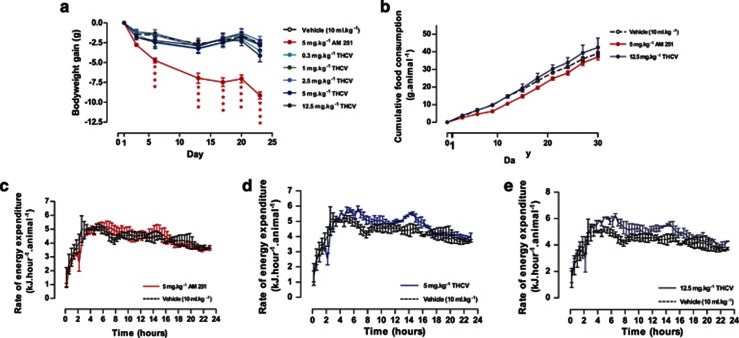
Effect of THCV on body weight gain, cumulative food intake and energy expenditure in DIO mice (study 1). (**a**) Body weight gain in the dose–response study, *n*=9 mice per treatment. (**b**) Cumulative food intake in mice given AM251 or THCV (12.5 mg kg^−1^ p.o.), *n*=3 groups of three mice per treatment (other dose levels of THCV excluded from graph for sake of clarity since identical to controls). However, all treatments were included in the one-way ANOVA statistical analysis. (**c**) Twenty-four-hour energy expenditure after 9 days treatment in DIO mice given AM251, (**d**) THCV (5 mg kg^−1^) or (**e**) THCV (12.5 mg kg^−1^ p.o.) in study 1, *n*=3 groups of three mice per treatment. ****P<0.0001 as compared to vehicle treated animals.

*Effect of THCV on body weight in DIO mice:* In study 1 in DIO mice (starting body weight 36.3±0.4 g), AM251 (5 mg kg^−1^ twice daily), a synthetic inverse agonist at CB1 receptors used as a positive control, reduced body weight by a mean of >8 g (*P*<0.001), whereas THCV at doses from 0.3 to 12.5 mg kg^−1^ twice daily had no significant effect on body weight (Figure 1a). In study 2 (starting body weight of mice 33.1±0.3 g), which used once per day dosing, AM251 (10 mg kg^−1^ p.o. daily) reduced body weight over the first 10 days and it then stabilized. Again THCV (0.1 –12.5 mg kg^−1^) had no significant effect on body weight (Supplementary Figure 1a).

*Effect on food and water consumption:* In study 1, there was no effect of THCV on food intake throughout the study. Whereas, although there was no overall effect on food intake with AM251, this compound did decrease the total food intake over the first 10 days of treatment —one-way ANOVA, *P*<0.01 (Figure 1b). Neither AM251 nor THCV affected water intake (data not shown).

*Effect on body composition:* Body composition was measured at the end of study 2 using dual X-ray absorptiometry. AM251-treated mice had a significantly lower fat content than controls (26.4±1.4% vs 42.1±1.7% *P*<0.01), and the two highest doses of THCV resulted in a less strong but nevertheless statistically significant reduction of body fat mass (35.1±1.4% and 31.1±1.2%, respectively; *P*<0.01 for both treatments relative to controls). There was no significant effect on lean body mass of any treatment.

*Energy expenditure:* In study 1, energy expenditure was measured by indirect calorimetry on days 9–10 and there was no effect of either AM251 (5 mg kg^−1^; Figure 1c) or THCV at doses of 0.3–2.5 mg kg^−1^ when data was expressed per mouse. However, at 5 and 12.5 mg kg^−1^ THCV tended to increase energy expenditure by 8.2% and 13.5%, respectively (Figures 1d and e) when this was expressed per mouse. When expressed per kg body weight, the energy expenditure of the AM251-treated mice was 13.2% greater than that of vehicle-treated controls (Supplementary Figure 1b) as, although the mice were smaller than controls, energy expenditure per mouse was similar to controls (Figure 1c). THCV at 5 and 12.5 mg kg^−1^ also tended to increase energy expenditure by 7.5% and 17.1%, respectively on a per kg body weight basis relative to controls (Supplementary Figure 1b).

In study 2, energy expenditure tended to be increased by AM251 (10 mg kg^−1^) and the two higher doses of THCV by >10% when results were expressed on an individual mouse basis (Supplementary Figure 1e).

**Figure 2:**
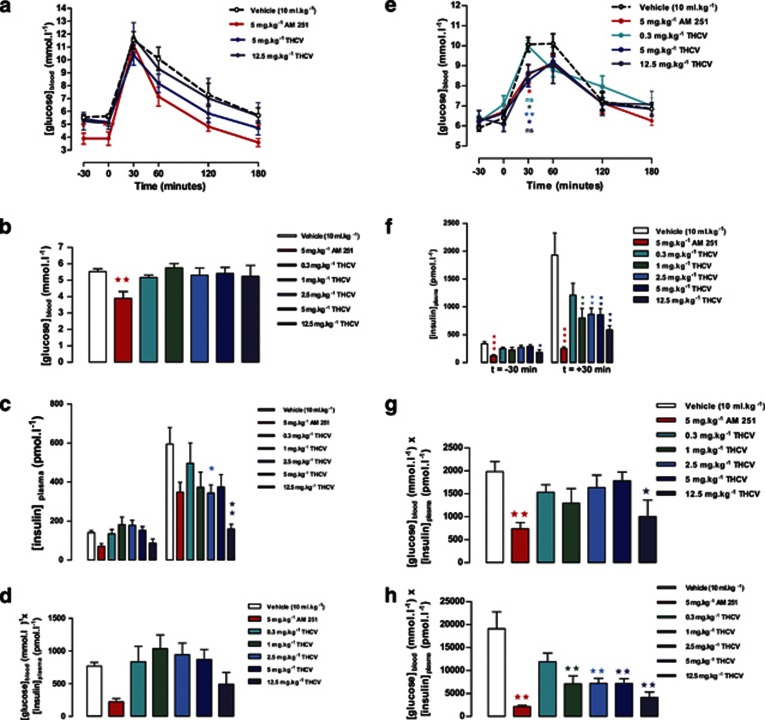
Effect of THCV on glucose tolerance and indexes of insulin sensitivity in DIO mice (study 1). (**a**) Glucose tolerance after 7 days treatment with AM251 or THCV, *n*=9 mice per treatment. (**b**) Blood glucose concentration in 5-h fasted mice after 7 days treatment with AM251 or THCV, *n*=9 mice per treatment. (**c**) Plasma insulin in 5-h fasted mice and at 30 min post glucose load after 7 days treatment with AM251 or THCV, *n*=9 mice per treatment. (**d**) Homeostatic model assessment (HOMA) index (glucose × insulin) in 5-h fasted mice after 7 days treatment with AM251 or THCV. (**e**) Glucose tolerance after 3 weeks treatment with AM251 or THCV, *n*=9 mice per treatment. (**f**) Plasma insulin in 5-h fasted mice at 30 min post glucose load after 3 weeks treatment with AM251 or THCV, *n*=9 mice per treatment. (**g**) HOMA index (glucose × insulin) in 5-h fasted mice after 3 weeks treatment with AM251 or THCV. (**h**) HOMA index (glucose × insulin) at 30 min post glucose load after 3 weeks treatment with AM251 or THCV. *P<0.05; **P<0.01 as compared to vehicle treated animals.

*Glucose tolerance and fasting glucose and insulin:* In study 1, after dosing for 7 days, AM251 improved glucose tolerance, as measured by OGTT (determined by area under blood glucose curve *P*<0.05), after 1 week of treatment (see time-course in Figure 2a). THCV had no significant effect on the blood glucose area under the time curve. AM251 also reduced the 5-h fasted glucose concentration (Figure 2b). However, both AM251 and THCV at the 12.5-mg kg^−1^ dose reduced the fasting insulin concentration (*P*<0.05; Student's *t*-test; Figure 2c, left panel). THCV resulted in a dose-related reduction in the insulin concentration measured 30 min after the glucose load with reduction following the 2.5- and 12.5-mg kg^−1^ dose being significant (*P*<0.01) (Figure 2c, right panel). Thus, although glucose tolerance was not affected by THCV, the glucose profile was achieved with a lower insulin concentration as shown also by the product of post glucose load blood glucose concentration and plasma insulin concentration, an index of tissue insulin sensitivity (Figure 2d).

The glucose tolerance test was repeated after 3 weeks dosage. AM251 (5 mg kg^−1^) and the two highest doses of THCV (5 and 12.5 mg kg^−1^) gave similar glucose tolerance profiles with significant reductions in glucose concentrations at 30 min post glucose load (Figure 2e). But, as noted after 1 week treatment, the major effect was on the 5-h fasted and post-glucose plasma insulin concentrations (Figure 2f). AM251 and THCV (12.5 mg kg^−1^) also improved the insulin sensitivity index (product of blood glucose concentration and plasma insulin concentration) in the fasted state (Figure 2g) and there was a dose-related effect of THCV in the fed state (Figure 2h).

In study 2, where the mice were given the treatments once daily, only the highest dose of THCV lowered the fasting glucose concentration (*P*<0.05; Supplementary Figure 2a) after 18 days of treatment, but the fasting insulin concentration was significantly reduced by AM251 (*P*<0.001) and by THCV in a dose-related manner (Supplementary Figure 2b). The insulin sensitivity index was similarly affected (Supplementary Figure 2c).

*Plasma lipids:* In study 1, AM251 increased both HDL cholesterol and total cholesterol significantly (*P*<0.05) in fed mice, but THCV had no effect and neither drug affected plasma triglyceride concentrations in fed mice (Supplementary Figures 3a–c). In study 2, total cholesterol concentration was significantly reduced by THCV at doses of 0.5, 2.5 and 12.5 mg kg^−1^ (Supplementary Figure 3d) but HDL cholesterol concentrations were unchanged (Supplementary Figure 3e). There was no treatment-related effect on plasma triglyceride concentration.

*Liver triglyceride content:* There was no significant effect of any treatment on liver triglyceride content in this model in either study 1 or study 2 (data not shown).

### Studies in *ob/ob* mice

#### Study 3 (pilot study with two doses of THCV) and study 4 (dose-ranging study)

**Figure 3:**
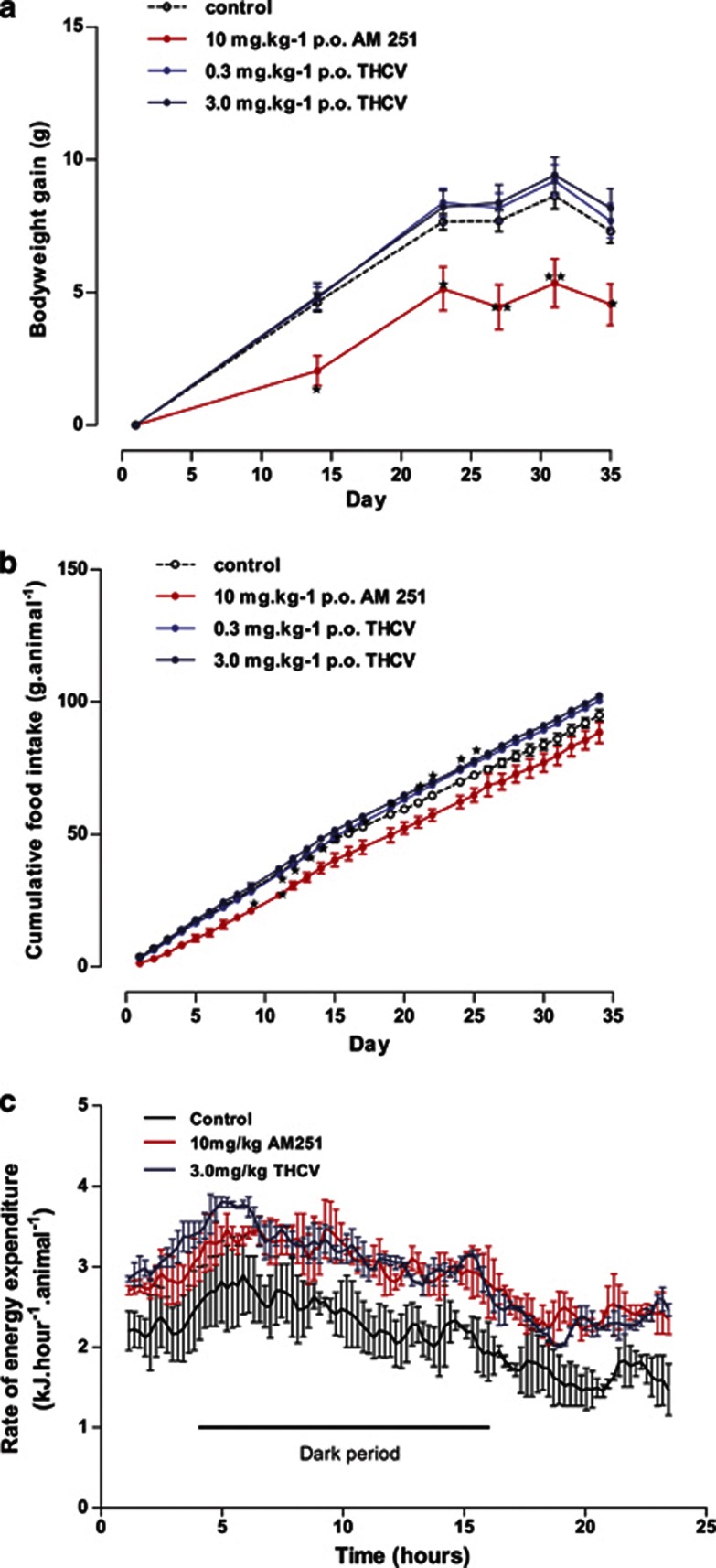
Effect of THCV on body weight gain, cumulative food intake and energy expenditure in *ob/ob* mice (study 3). (**a**) Body weight gain in mice given AM251 or THCV, *n*=8 mice. (**b**) Cumulative food intake in mice given AM251 or THCV, *n*=2 groups of four mice per treatment. (**c**) Twenty-four-hour energy expenditure in mice given AM251 or THCV, *n*=2 groups of four mice per treatment. *P<0.05; **P<0.01 as compared to vehicle treated animals.

*Body weight gain and food intake:* In a pilot study in *ob/ob* mice (study 3), THCV (3 mg kg^−1^) had no effect on body weight or body weight gain, whereas AM251 (10 mg kg^−1^) reduced body weight and body weight gain significantly (Figure 3a). Similarly, THCV had no effect on food intake, whereas AM251 reduced food intake over the first few days of treatment (Figure 3b). Having demonstrated an effect of AM251 in the pilot study, study 4 examined the effect of THCV over the dose-range of 0.1–12.5 mg kg^−1^. There was no effect of THCV on body weight gain or food intake over this dose range (Supplementary Figures 4a and b).

*Energy expenditure:* In the pilot study, energy expenditure was measured after 10 days of treatment. Both AM251 (10 mg kg^−1^) and THCV (3 mg kg^−1^) increased the 24-h energy expenditure by around 30% (Figure 3c). This effect was independent of whether the data was expressed per mouse or per kg body weight (data not shown).

**Figure 4:**
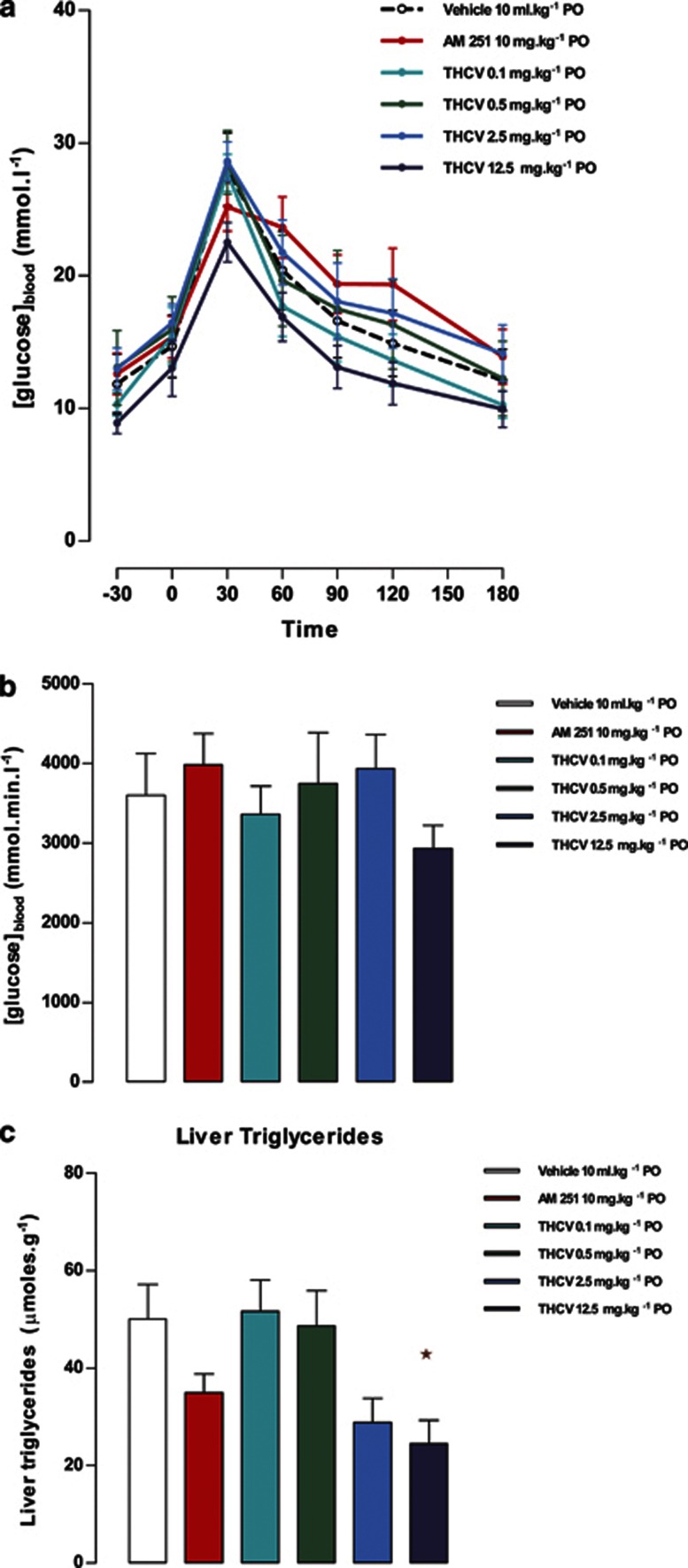
Effect of THCV on glucose tolerance and liver triglycerides in *ob/ob* mice (study 4). (**a**) Glucose tolerance after 3 weeks treatment with AM251 or THCV, *n*=8. (**b**) Area under the glucose tolerance curve, *n*=8. (**c**) Liver triglycerides content after 4 weeks treatment with AM251 or THCV, *n*=8. *P<0.05 as compared to vehicle treated animals.

*Effects on fasting glucose and insulin, and glucose tolerance:* Glucose tolerance was measured after 3 weeks of treatment in study 4. There was no significant effect of any treatment on the blood glucose concentration or the plasma insulin concentration after a 5-h fast (data not shown). Glucose tolerance was not significantly unaffected either by AM251 (10 mg kg^−1^) or by THCV (12.5 mg kg^−1^) (Figures 4a and b).

*Plasma lipids:* There was no effect of any dose of THCV on fed plasma total cholesterol, HDL cholesterol or triglyceride levels after 4 weeks of treatment (data not shown).

*Liver fat and glycogen:* The highest doses of THCV (2.5 and 12.5 mg kg^−1^) produced an ∼50% reduction in liver triglyceride concentration, an effect that was statistically significantly for the 12.5-mg kg^−1^ dose (Figure 4c). AM251 also reduced liver triglycerides but in a non-statistically significant manner. Liver glycogen was unaffected by treatment (data not shown).

**Figure 5:**
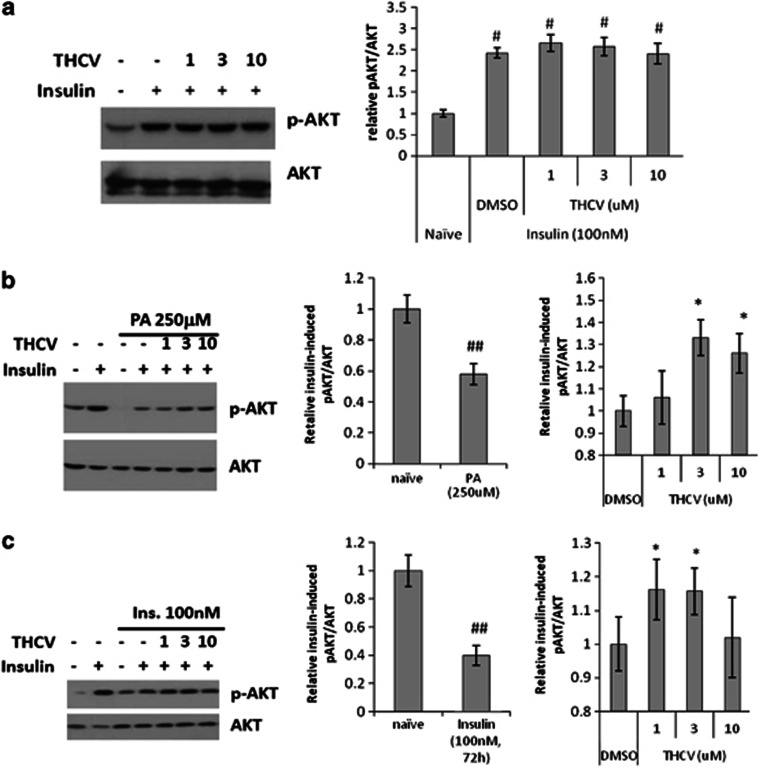

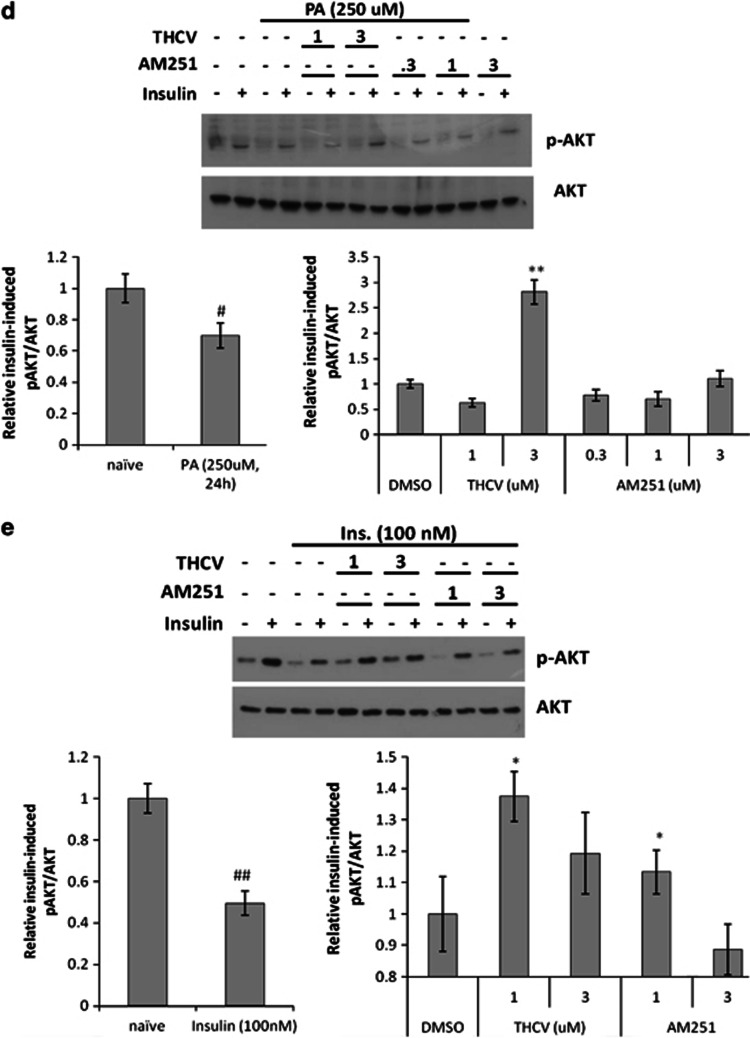
Effect of THCV on insulin-induced phosphorylation of Akt in insulin-resistant human HHL-5 hepatocytes. (**b**, **c**) HHL-5 cells were rendered insulin-resistant following 72 h incubation with 100 nℳ insulin (Ins.) (**b**) or 48 h incubation with 0.25 mℳ palmitic acid (PA) (**c**). A representative western blot for insulin stimulation of Akt phosphorylation in insulin-sensitive cells is shown in (**a**), the right panel indicating the fold-stimulation by insulin of basal phosphoAkt (pAKT)/total Akt (AKT), quantified by densitometry in absence (DMSO) or presence of THCV 1–10 μℳ. (**b**, **c**) Representative western blots for insulin stimulation of Akt phosphorylation in insulin-resistant hepatocytes incubated for the final 24 h of chronic insulin or PA incubation with the indicated concentrations of THCV. The middle panels, obtained by densitometry quantification of *n*=3 separate western blots, indicate the lower stimulation by acute insulin of pAKT/AKT levels in desensitized cells as compared to insulin-sensitive cells (naïve), considered as 1. The right panels indicate the effect of THCV on insulin-induced stimulation of pAKT/AKT levels in insulin-resistant cells as compared with insulin-resistant cells only treated with acute insulin and THCV vehicle (DMSO), considered as 1. (**d**, **e**) Representative western blots for insulin stimulation of Akt phosphorylation in hepatocytes made insulin-resistant with a 24-h treatment with insulin or PA and co-incubated with the indicated concentrations of THCV, AM251 or vehicle. The middle panels, obtained by densitometry quantification of *n*=3 separate western blots, indicate the lower stimulation by acute insulin of pAKT/AKT levels in desensitized cells as compared with insulin-sensitive cells (naïve), considered as 1. The right panels indicate the effect of THCV or AM251 on insulin-induced stimulation of pAKT/AKT levels in insulin-resistant cells as compared with insulin-resistant cells only treated with acute insulin and vehicle (DMSO), considered as 1. ^#^*P*<0.05 or ^##^*P*<0.01 vs naïve. *P<0.05 or **P<0.01 vs DMSO, as assessed by ANOVA followed by the Bonferroni's test.

*Effect of THCV on insulin signalling in insulin-resistant human hepatocytes:* In insulin-sensitive cells, insulin efficiently stimulated Akt phosphorylation, whereas THCV had no additional effect on Akt activation in this state (Figure 5a). In ‘reversal' experiments, human HHL-5 hepatocytes were rendered insulin-resistant following 48 h incubation with 250 μℳ palmitic acid or 72 h incubation with 100 nℳ insulin, the final 24 h of which included co-incubation with 1, 3 and 10 μℳ THCV. In these cells, 100 nℳ insulin did not efficiently stimulate Akt, however, incubation with 3 or 10 μℳ THCV (in palmitic acid-pretreated cells) and 1 or 3 μM THCV (in insulin-pretreated cells) increased the sensitivity of HHL-5 cells to insulin in terms of Akt activation (Figures 5b and c). When co-incubated with palmitic acid or insulin (‘prevention' experiments), THCV enhanced insulin-induced Akt phosphorylation at 3 and 1 μℳ respectively (Figures 5d and e). In contrast, AM251 was able to only partly sensitize cells to insulin in the presence of chronic insulin, but not palmitic acid (Figures 5d and e).

**Figure 6:**
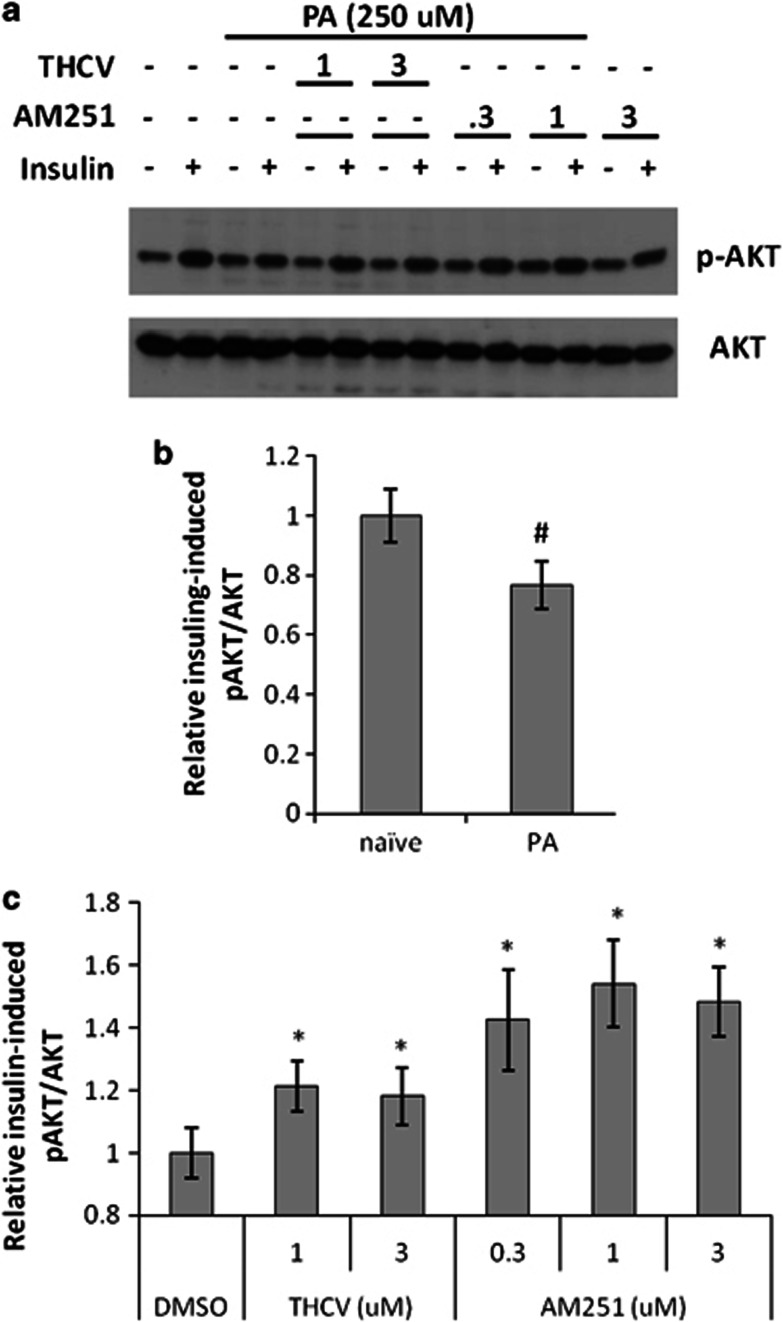
Effect of THCV on insulin-induced phosphorylation of Akt in insulin-resistant rat C_2_C_12_ myotubes. Differentiated C_2_C_12_ cells were rendered insulin-resistant following 24 h incubation with 0.25 mℳ palmitic acid (PA) and co-treated with either THCV, AM251 or vehicle, at the concentrations shown. (**a**) Representative western blot for insulin stimulation of Akt phosphorylation in insulin-resistant cells. (**b**) Densitometric quantification of *n*=2 separate western blots, indicating the lower stimulation by acute insulin of pAKT/AKT levels in desensitized cells as compared to insulin-sensitive cells (naïve), considered as 1. (**c**) Effect of THCV or AM251 on insulin-induced stimulation of pAKT/AKT levels in insulin-resistant cells as compared to insulin-resistant cells only treated with acute insulin and vehicle (DMSO), considered as 1. ^#^*P*<0.05 vs naïve. *P<0.05 vs DMSO, as assessed by ANOVA followed by the Bonferroni's test.

*Effect of THCV on insulin signalling in insulin-resistant mouse C_2_C_12_ myotubes:* In insulin-sensitive differentiated C_2_C_12_ myotubes, insulin efficiently stimulated Akt phosphorylation (Figure 6a). When myotubes were treated for 24 h with 250 μℳ palmitic acid, they responded significantly less to acute insulin (by about 25% Figures 6a and b), but co-incubation with THCV (1 and 3 μℳ) throughout the desensitization period restored insulin sensitivity (Figures 6a and c). AM251 (0.3–3 μℳ) appeared to more significantly rescue insulin stimulation of Akt phosphorylation (Figures 6a and c).

## Discussion

This study is the first to investigate the effects of chronic administration of THCV in animal models of obesity, and to demonstrate the positive metabolic effects of this plant cannabinoid in obese mice with metabolic disturbances. In agreement with the original report of THCV as a CB1 neutral antagonist, which would be possibly safer with regard to potential psychiatric side effects than CB1 inverse agonists, we show here that this compound produces effects in obese mice that are both qualitatively and quantitatively different from those of a widely used CB1 inverse agonist, AM251.

In DIO mice, we observed that, like AM251, THCV dose-dependently improved fasting plasma glucose and glucose tolerance following an OGTT and, especially when administered twice daily, improved insulin sensitivity in terms of fasting plasma insulin and insulin response to an OGTT. Furthermore, THCV, again like AM251, increased energy expenditure, particularly in *ob/ob* mice. On the other hand, in *ob/ob* mice, both THCV and AM251 were less efficacious at reducing glucose intolerance, but THCV was more efficacious than AM251 at reducing liver triglyceride levels. Perhaps more importantly, the effects of THCV, unlike those of AM251, were never accompanied by significant reduction of food intake or body weight gain, and, in this sense, this compound also differs from CB1 neutral antagonists, which instead do exert these actions. Finally, we found that THCV, like AM251, improves insulin-induced phosphorylation of Akt in hepatocytes and myotubes made insulin-resistant by prolonged treatment with insulin or palmitic acid.

It is important to review these findings in light of the effects previously reported for CB1 inverse agonists or neutral antagonists in *in vivo* models of obesity and type 2 diabetes. It is now generally accepted that peripheral overactivity of the endocannabinoid system is linked to abdominal obesity and other risk factors for cardiovascular disease and type 2 diabetes.^16^ It is more than 10 years since the first report of Rimonabant reducing food intake and body weight in *ob/ob* mice with elevated hypothalamic endocannabinoid levels was published.^17^ Ravinet Trillou *et al.*^18^ later reported that, in DIO mice, a 5-week treatment with Rimonabant (10 mg kg^−1^ per day orally) induced a transient reduction of food intake (−48% on week 1) and a marked but sustained reduction of body weight (−20%) and adiposity (−50%). Furthermore, Rimonabant corrected the insulin resistance and lowered plasma leptin, insulin and free fatty acid levels. Most of these effects: (i) could be dissociated from the food-intake inhibitory, but not the body weight-lowering, effects of the drug; (ii) were present, but less pronounced, with the lower dose used (3 mg kg^−1^ per day); and (iii) were confirmed in subsequent studies with different rat and mouse genetic or dietary models of obesity as well as in dogs fed a high-fat diet.^16^ Subsequent work showed that the anti-obesity effect of Rimonabant was also associated with an improved serum lipid profile, again in a manner seemingly independent from the anorexic actions of the drug.^19^ Rimonabant treatment (10 mg kg^−1^ per day for 10 weeks) significantly reduced the high-fat diet-induced elevations in leptin, insulin and glucose, and modestly but significantly increased serum adiponectin levels. Although it did not modify HDL cholesterol, it had modest effects on total cholesterol and significantly reduced triglycerides and LDL cholesterol, thus increasing the HDL/LDL ratio. Soon thereafter, the effect of 1-year treatment with Rimonabant on weight reduction and cardiovascular risk factors in obese patients (RIO-Europe) was published.^20^ In this clinical study it was observed that weight loss at 1 year was significantly greater in patients on a mild hypocaloric diet treated with Rimonabant 20 mg compared with placebo, and that this dose of the compound produced significant improvements in waist circumference, HDL cholesterol, triglycerides and insulin resistance, and reduced the prevalence of the metabolic syndrome as compared with placebo. This study represented evidence that the beneficial metabolic effects of CB1 inverse agonists can be achieved in humans, and was followed by several other successful phase III trials with Rimonabant and other compounds in the same class in obese subjects with dyslipidaemia or type 2 diabetes.

Interestingly, the improvement of liver triglyceride levels observed here with oral THCV (12.5 mg kg^−1^) in *ob/ob* mice (in which there is defective leptin production) is comparable to the effect in obese Zucker (*fa/fa*) rats (which are impaired in leptin action) of daily oral Rimonabant (30 mg kg^−1^) for 8 weeks. This treatment also reduced parameters that were not monitored here, that is, hepatomegaly and plasma levels of enzyme markers of hepatic damage. Indeed, the finding that THCV is able to reduce liver triglyceride levels is unsurprising as activation of hepatic CB1 receptor is instead sufficient for the development of diet-induced steatosis, dyslipidaemia and insulin resistance in mice.^21^ More recently, selective CB1 activation in hepatocytes was reported to cause hepatic insulin resistance,^22^ and, accordingly, we have shown here that THCV instead restores insulin sensitivity in these cells after that they were rendered insulin-resistant.

In Zucker rats, Rimonabant also reduced plasma tumour necrosis factor-alpha levels, increased the plasma adiponectin levels and improved dyslipidaemia by decreasing plasma levels of triglycerides, free fatty acids and total cholesterol, and increasing the HDL/LDL ratio. These two latter effects were also observed here with the highest tested dose of THCV, but only in DIO mice and, quite interestingly, with the once a day, and not with the twice daily, administration protocol. On the other hand, administering the same dose of THCV once or twice daily to DIO mice did not appear to make an overall great difference in terms of reducing fasting glucose or glucose intolerance. However, after a 3-week treatment, the once daily administration of the drug did lower fasting glucose, whereas the twice daily administration of THCV did reduce glucose intolerance, in a statistically significant manner. In both cases, THCV appeared to affect fasting insulin and insulin levels after OGTT more profoundly than fasting glucose or glucose intolerance. THCV appears to differ in its action from Rimonabant and other inverse agonists in that it had no significant effect on glucose tolerance in *ob/ob* mice suggesting that its effect on glucose tolerance might be leptin-dependent.

Interestingly, and contrary to what was observed here with AM251 and previously with Rimonabant,^23^ the increased energy expenditure induced by THCV in the present studies never resulted in any body weight-reducing effect. However, in the study in which the compound was administered twice a day to DIO mice a significant dose-dependent reduction in body fat mass was observed. Indeed, increased energy expenditure might produce beneficial effects on glucose tolerance and insulin resistance also in the absence of overt changes in body weight, as observed for example when CB1 receptors are selectively deleted in hepatocytes.^21^

In summary, although THCV, which, like THC and AM251, has high affinity for CB1 receptors and high brain penetration,^12, 24^ did produce some of the metabolically beneficial effects typical of CB1 inverse agonists, its actions in obese mice differed from those of a comparable dose of AM251, particularly as they were observed in the absence of any effect on body weight. In this respect, THCV behaved in different manner also as compared with synthetic CB1 neutral antagonists.^2^ The mechanism(s) through which THCV exerts these differential effects remain(s) to be fully elucidated. For instance, it has been reported that CB1 receptor blockade promotes mitochondrial biogenesis through endothelial nitric oxide synthase expression in white adipocytes,^25^ and it would be interesting to investigate if THCV is capable of similar effects *in vitro*, as such effects were suggested to underlie Rimonabant-induced lipid diversion from storage towards utilization and its reduction of fat mass.^26^ On the other hand, the counteraction of chronic insulin- and palmitic acid-induced impairment of insulin receptor signalling in hepatocytes, reported here for THCV, suggests that the effect of this compound on glucose tolerance in obese mice might be partly due to antagonism of CB1-induced impairment of insulin inhibition of hepatic glycogenolysis.^22^ Yet, it remains to be seen whether chronic CB1 antagonism in insulin-resistant peripheral organs is the main mechanism of action for THCV, or whether there are other additional molecular targets contributing to its therapeutic benefits. For example, this compound was reported to antagonize or activate CB2 receptors depending on its concentrations.^10, 11, 12^ Furthermore, THCV is also known to activate TRPV1 channels,^27^ which were recently implicated in the restoration of insulin sensitivity in DIO mice by capsaicin.^28^

In conclusion, THCV produces therapeutic metabolic effects in two different mouse models of obesity. In particular, its strongest effects are exerted on plasma glucose and insulin levels, especially following an OGTT in DIO mice and on liver triglycerides in *ob/ob* mice. Based on these data, it can be suggested that THCV may be useful for the treatment of the metabolic syndrome and/or type 2 diabetes, either alone or in combination with existing treatments. Given the reported benefits of another non-THC cannabinoid, CBD in type 1 diabetes,^29, 30^ a CBD/THCV combination may be beneficial for different types of diabetes mellitus.

## Notes

This work was supported by a grant from GW Pharmaceuticals to MA Cawthorne and V Di Marzo. GW Guy, CG Stott and M Duncan are salaried employees of GW Pharmaceuticals. GW Guy and CG Stott are stockholders in GW Pharmaceuticals.
