# Supplementary figures and images for: The cannabinoid Δ9-tetrahydrocannabivarin (THCV) ameliorates insulin sensitivity in two mouse models of obesity
Source: Nutr Diabetes. 2013 May 27;3(5):e68–. doi: 10.1038/nutd.2013.9 (PMC3671751; doi:10.1038/nutd.2013.9)

**a**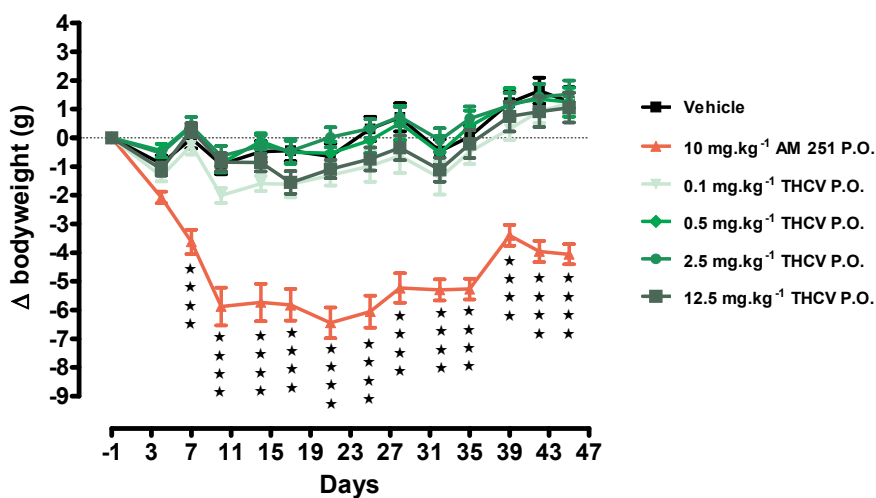**b**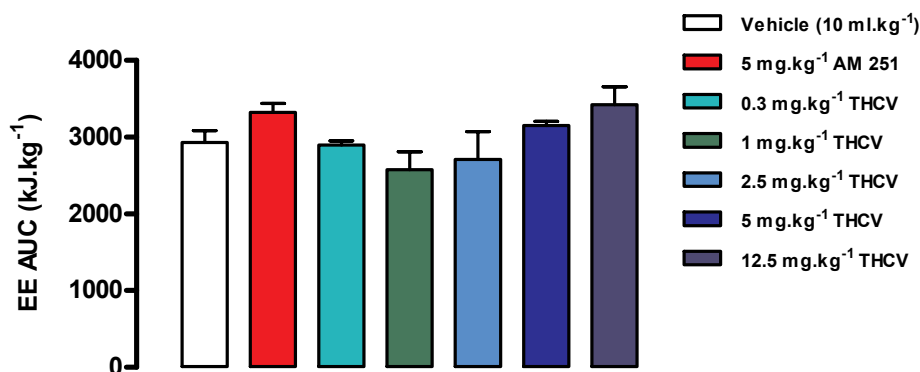**c**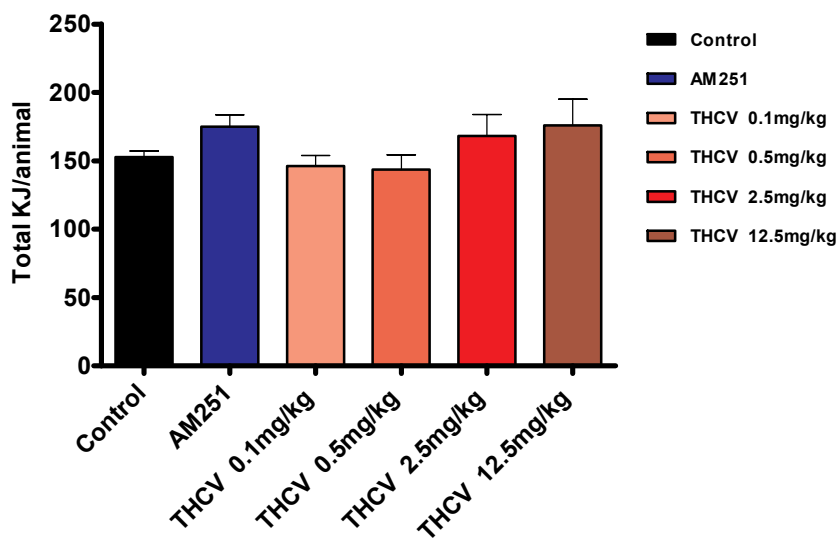

Supplement: Supplementary Figure 1 [file nutd20139x1.pdf]

**a**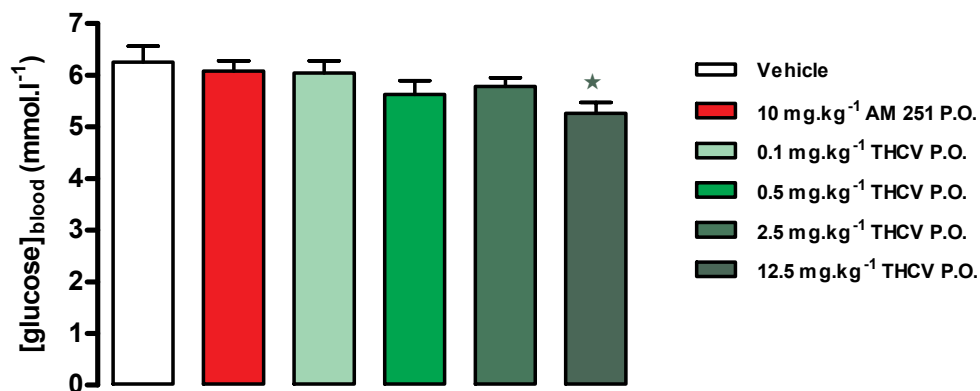**b**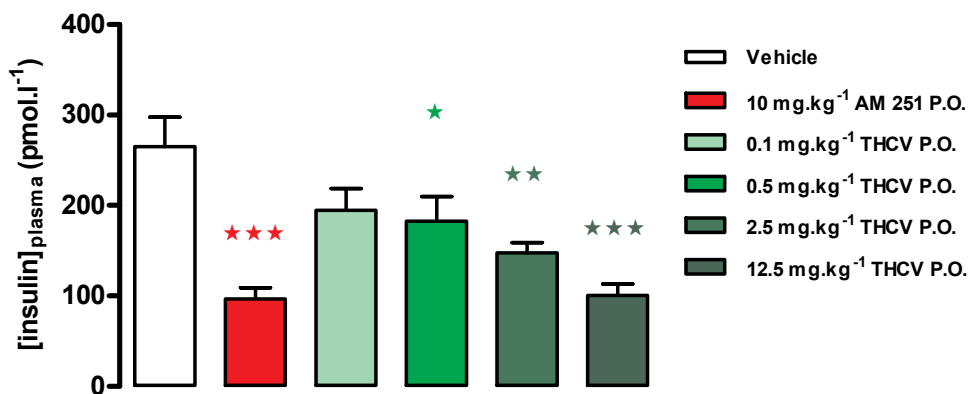**c**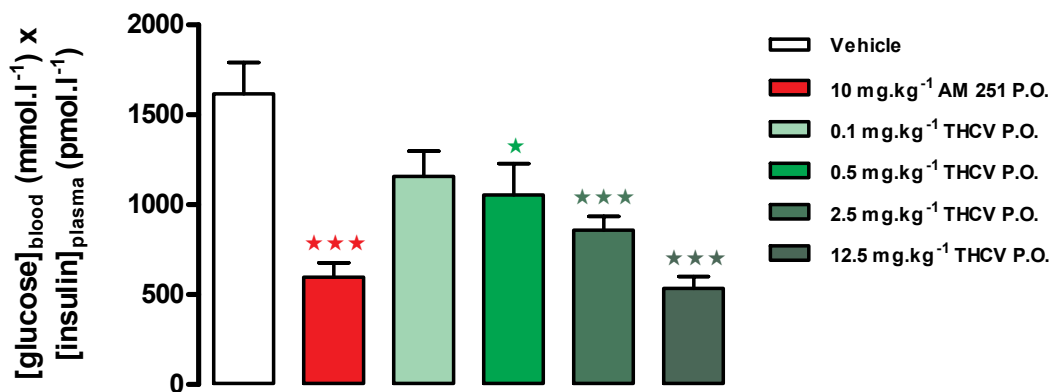

Supplement: Supplementary Figure 2 [file nutd20139x2.pdf]

**a**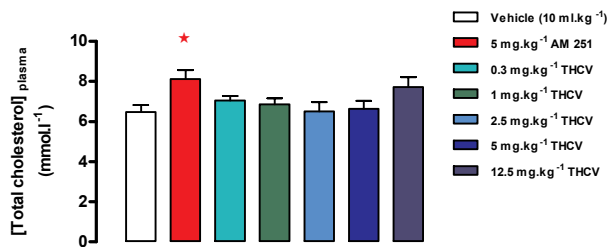**d**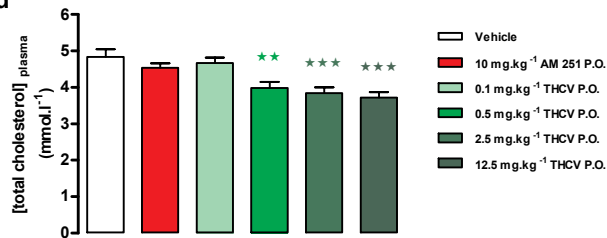**b**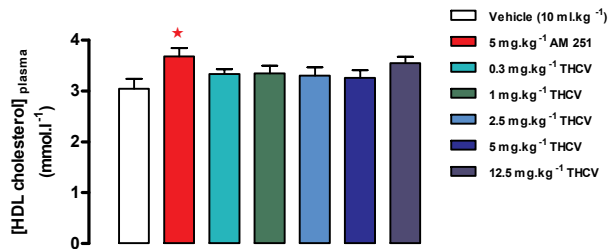**e**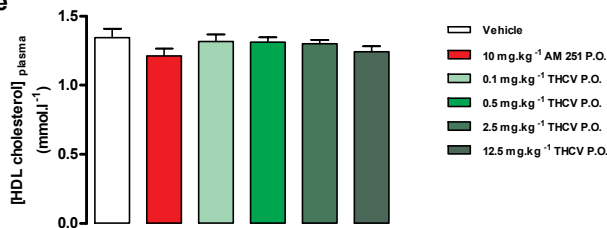**c**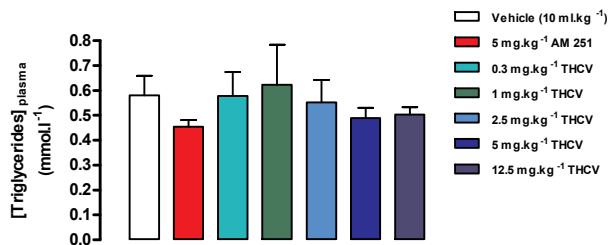

Supplement: Supplementary Figure 3 [file nutd20139x3.pdf]

a

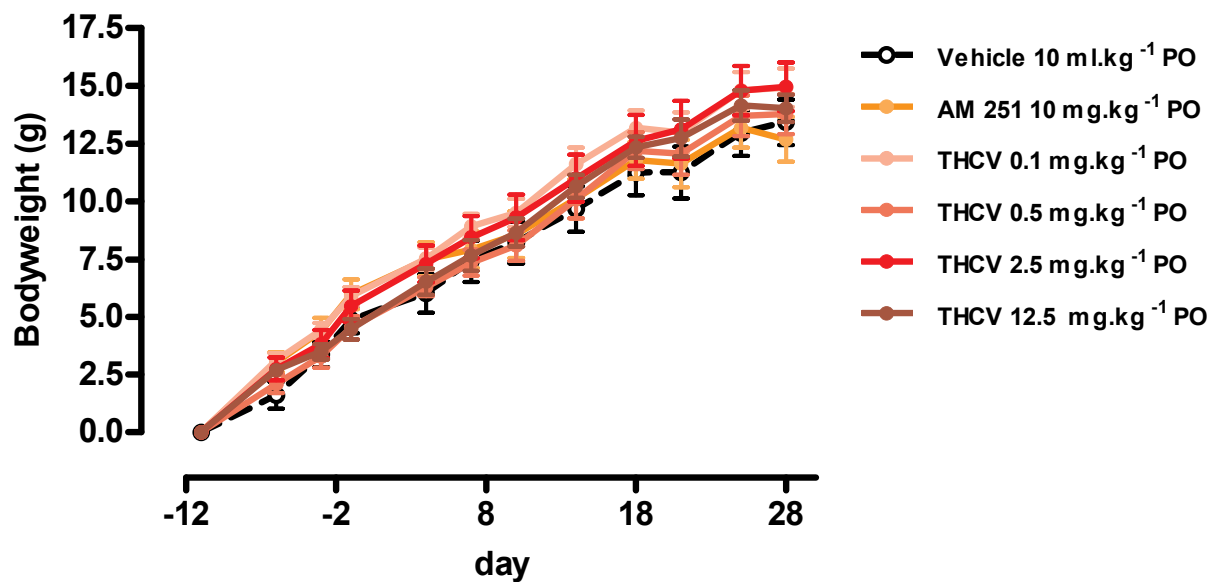

b

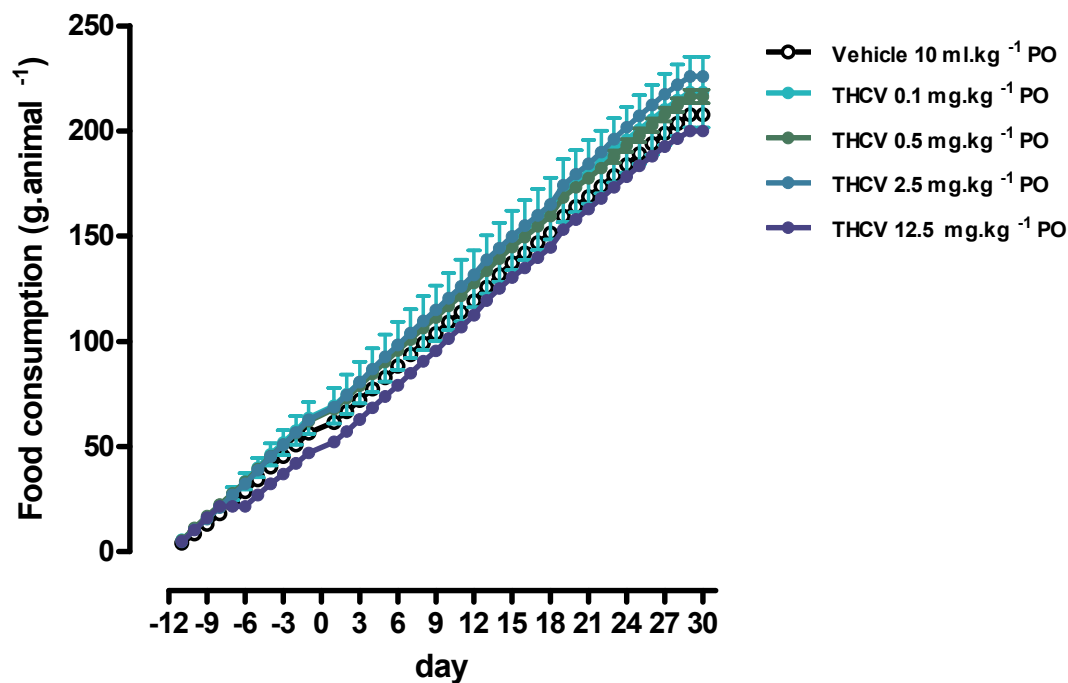

Supplement: Supplementary Figure 4 [file nutd20139x4.pdf]
