# Supplementary material for: The cannabinoid Δ9-tetrahydrocannabivarin (THCV) ameliorates insulin sensitivity in two mouse models of obesity
Source: Nutr Diabetes. 2013 May 27;3(5):e68–. doi: 10.1038/nutd.2013.9 (PMC3671751; doi:10.1038/nutd.2013.9)
Supplement: Supplementary Information [file nutd20139x5.doc]

Supplementary Figure 1. Dose-response study of THCV and AM251 (10 mg/kg) on body weight and energy expenditure in DIO mice in studies 1 and 2. (a) Body weight change in DIO mice treated for 4 weeks with AM251 or THCV in study 2, n = 9. (b) Energy expenditure of DIO mice after 9 days treatment expressed as area under the curve kg body wt-1 in study 2, n = 3 groups of 3 mice per treatment. (c) Energy expenditure of DIO mice in THCV dose response study after 10 days treatment expressed as area under the curve per mouse in study 2, n = 3 groups of 3 mice per treatment.

Supplementary Figure 2. Dose-response study of THCV and AM251 (10 mg/kg) on fasting glucose and insulin and HOMA-1R in DIO mice in study 2. (a) Blood glucose concentration in 5h-fasted DIO mice after 3 weeks treatment with AM251 or THCV in study 2, n = 9. (b) Plasma insulin concentration in 5h-fasted DIO mice after 3 weeks treatment with AM251 or THCV in study 2, n = 9. (c) Insulin sensitivity index (fasting glucose x insulin) in DIO mice after 3 weeks treatment with AM251 or THCV in study 2, n = 9.

Supplementary Figure 3. Effect of THCV and AM251 on plasma total cholesterol, HDL cholesterol and triglyceride in DIO mice in studies 1 and 2. (a) Plasma cholesterol concentration in DIO mice after 3 weeks treatment with AM251 or THCV in study 1, n = 6-9 mice. (b) Plasma HDL-cholesterol concentration in DIO mice after 3 weeks treatment with AM251 or THCV in study 1, n = 6-9 mice. (c) Plasma triglyceride concentration in DIO mice after 3 weeks treatment with AM251 or THCV in study 1, n = 6-9 mice. (d) Plasma cholesterol concentration in DIO mice after 4 weeks treatment with AM251 or THCV in study 2, n = 9. (e) Plasma HDL-cholesterol concentration in DIO mice after 4 weeks treatment with AM251 or THCV – study 2, n = 9.

Supplementary Figure 4. Effect of THCV on body weight and food intake in *ob/ob* mice in study 4. (a) Body weight gain in *ob/ob* mice given THCV in study 4, n = 8. (b) Cumulative food consumption in *ob/ob* mice given 28 THCV in study 4, n = 2 groups of 4 mice per treatment.
